# The Impact of Residual Symptoms in Major Depression

**DOI:** 10.3390/ph3082426

**Published:** 2010-08-03

**Authors:** Joshua A. Israel

**Keywords:** major depressive disorder, MDD, antidepressive agents, residual symptoms, depression, depression treatment

## Abstract

The current definition of remission from major depressive disorder does not fully take into account all aspects of patient recovery. Residual symptoms of depression are very common in patients who are classified as being in remission. Patients with residual symptoms are at increased risk of functional and interpersonal impairments, and are at high risk for recurrence of depression. This article discusses the incidence of residual symptoms of depression, as well as the risks and consequences of these symptoms, and will review the state of current treatment.

## 1. Introduction

It is well established that approximately 55% of patients with Major Depressive Disorder (MDD) will respond to treatment with an initial antidepressant medication [1]. “Response,” when considered in its colloquial meaning, sounds like a highly desirable treatment outcome for a patient with depression. In the psychiatric literature, however, this word has a precise meaning; patients and clinicians alike should have an understanding of this meaning, so as to avoid inflated expectations and disappointment for patients and overconfidence and lack of close follow up on the part of clinicians. *Response* is defined as a 50% or more reduction in level of presenting symptomatology, as typically measured using a standardized rating scale, such as the Hamilton Depression Rating Scale (HAM-D) or the Montgomery Asberg Depression Rating Scale (MADRS) [2,3]. When the specific meaning of “response” is considered, it becomes clear that many patients may still be suffering greatly from symptoms of MDD, even when they can be considered treatment *responders*. As an example, a patient whose HAM-D score reduces from 32 to 16 with treatment will still likely be experiencing considerable impairment and distress. In some patients, *statistically* significant reductions in scores can even be demonstrated in the absence of *clinically* meaningful improvements [4]. 

**Figure 1:**
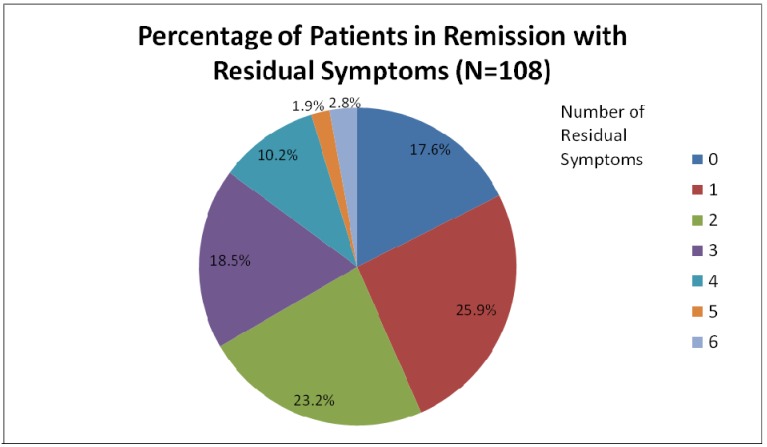
Patients in considered to be in remission from major depression who still had ongoing residual symptoms.

*Remission*, on the other hand, is conceptualized as a return to a state of normal functioning and minimal symptomatology [5]. Remission has been operationalized in clinical trials as a threshold, or cut-off score, using standardized scales. A HAM-D17 score of seven or less, a MADRS score of ten or less, or a Clinical Global Impression (CGI) score of one, all typically designate a state of remission [6]. Importantly, these criteria do not require that patients be completely asymptomatic to be considered in remission [3,7]. Even so, only 30% of patients in most clinical trials of antidepressant monotherapy achieve this limited state of remission [1]. In one study of 108 patients who had achieved remission, 26% had one residual symptom, and 57% had 2 or more symptoms [15] (Figure 1).

**Figure 2:**
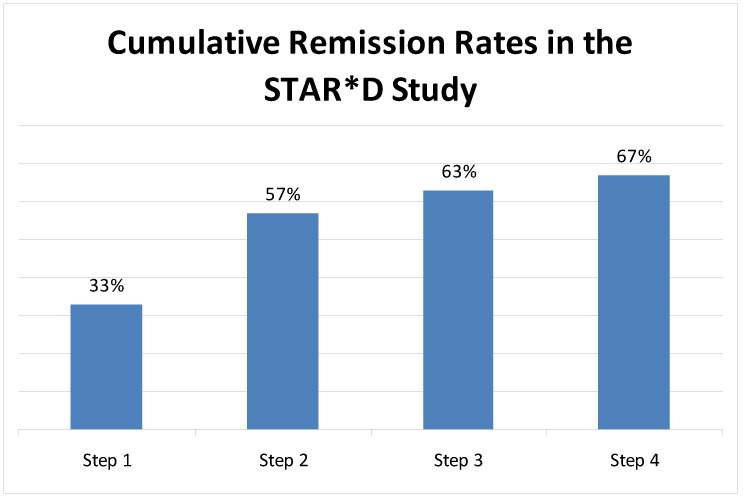
Cumulative remission rates at successive steps in the STAR*D study.

In the Sequenced Treatment Alternatives to Relieve Depression (STAR*D) study, 33% achieved remission after initial treatment with a selective serotonin reuptake inhibitor (SSRI). By the end of the study, 67% of patients had achieved remission, but this required four successive treatment steps of medications and psychotherapy [8] (Figure 2).

The actual cut-off score between response and remission is somewhat arbitrary [9], and depressed patients themselves may consider symptom resolution a less important indicator of remission than features such as optimism and self-confidence [10]. Nonetheless, the central notion is valid: it recognizes that treatment “response” is not a homogenous state and that there can be conceptual and practical benefits to separating out those who have differing levels of recovery, improvement in psychosocial function and risk of relapse. There are significant differences between treatment *responders* and *remitters* that do support such categorizations [11]. However, even among patients considered to be in remission, there can be considerable variability and heterogeneity in ongoing levels of impairment and function [12]. 

Patients whose MDD has been successfully treated to a state of remission can be further subclassified based on the presence of residual symptoms and the level of impairment of psychosocial functioning. One category includes patients who can be categorized as having achieved remission from MDD but continue to exhibit both residual symptoms and some functional impairment. A second category identifies patients in remission with no evident ongoing core depressive symptoms, but with the presence of some impairment in psychosocial functioning. The optimal category of remission is, of course, the complete absence of both depressive symptoms and functional impairments [2]. This optimal state of remission is unfortunately a very challenging target to achieve. As few as 12–18% of patients with MDD treated with an initial antidepressant may improve such that they can be classified as being completely free of residual depressive symptoms and functional impairments [13,14,15]. These subclassifications of remission more clearly describe patient status, and the distinctions have therapeutic implications [3]; patient prognosis and the variability of outcomes in treatment remission depend primarily on the degree of remission and level of residual symptomatology [3,12]. 

For many patients whose MDD is not in complete remission at the end of a clinical trial, it may simply be that the trial assessed patients at a 6–8 week endpoint; it is known that some patients take up to 12 weeks for a more complete remission [16]. For many patients, full recovery from MDD is an even more extended process than this [17], and residual symptoms can persist for many months after an episode of MDD can be classified as remitted [18]. 

Even so, a substantial body of literature demonstrates that among patients with MDD who meet remission criteria, those with residual symptoms have a significantly higher risk of relapse compared to those without residual symptoms [17,19]. The presence of residual symptoms is the single most accurate marker for risk of relapse back into full MDD [20,21,22,23]. In one large cohort study, patients without residual symptoms of depression relapsed at an average of 157 weeks, while those with residual symptoms relapsed in approximately 28 weeks [19]. Another study followed patients in remission from MDD for 10 months and found that 76% of patients with residual symptoms relapsed, compared to only 25% of those who were symptom-free [21]. Overall, patients with residual symptoms appear to be three times more likely to relapse into MDD than patients who are in complete remission [22]. Patients with residual symptoms are also more likely to experience a chronic course of illness [24], decreased likelihood of recovery over time, and increased psychosocial and socio-economic impairment [3]. 

## 2. Common Residual Symptoms

Among the most frequent residual symptoms of MDD are ongoing low mood, guilt, insomnia, anxiety, impaired work, loss of interest, irritability, fatigue, lowered libido, and a range of somatic or physical symptoms. Common residual somatic symptoms include backache, muscle ache, stomach aches and joint pain [14,25,26,27]. In addition to physical and emotional residual symptoms, cognitive deficits may remain in patients who otherwise appear to have reached remission, such as impaired memory processes and increased cognitive reactivity [28]. Residual symptoms are a predictor of relapse regardless of whether the patient was treated with medications or with psychotherapy [23]. 

The residual symptoms of insomnia and other sleep disturbances are independent predictors of recurrence of MDD [29,30,31]. Both self-report by patients and EEG sleep abnormalities can identify insomnia that may lead to the increased risk [32,33]. Insomnia as a residual symptom is also associated with poor response to treatment [34] and even risk of suicide [35]. Patients with premorbid anxiety have higher rates of recurrence and take longer to respond to treatment [36], and when anxiety is present as a residual symptom, this is also a predictor of more rapid recurrence of MDD, possibly even more so than any residual core mood symptoms [37]. In some patients, persistent anxiety may not resolve fully with remission of MDD because it represents a separate subsyndromal Axis I anxiety disorder [38,39], rather than a manifestation of persistent MDD. Many residual symptoms, including anxiety, are also frequently prodromal symptoms in the same patients [13,40], and as such can be useful as harbingers of recurrence of MDD. 

Several factors may help identify which patients will experience residual symptoms, though none are definitively predictive [41]. These include severity of the initial symptoms [21], the presence of dysthymia (“double depression”) [42], ongoing life stressors [27], medical illness burden [14], socioeconomic disadvantage [43], poor social supports [14] and inadequate treatment [44]. Residual symptoms cannot be predicted with accuracy by a patient’s age, sex, marital status, number of prior episodes or duration of the current episode [41]. There is mixed data as to whether duration of a major depressive episode is predictive of residual symptoms [19,41,43]. It may be that residual symptoms predict earlier time to relapse but not the overall long term risk of recurrence or number of recurrences [44]. One difficulty in determining the factors predictive of residual symptoms is that the predictive value may depend in each patient on what these residual symptoms represent. In some cases these symptoms will be represent separate, untreated disorders, while in other cases they are signs that the primary mood disorder is still present, and it is the untreated mood symptoms that are the higher risk category [22].

While many residual symptoms are best understood as a form of partial continuation of the index episode of depression, residual symptoms of MDD often include interpersonal difficulties, dysfunctional attitudes and cognitive distortions [45,46]. It is not clear if these symptoms are best categorized as *trait* or *state* dependent: are these stable, premorbid personality characteristics, or rather symptoms of depression that can resolve when MDD is entirely resolved? There is evidence to support both of these positions [47,48]. It seems realistic to presume that in some patients recovered from MDD, residual social maladjustment is the result of unfortunate but stable dysfunctional attitudes; in others these difficulties can improve with further treatment targeted to MDD, and in many there will be some combination thereof. 

There are many consequences to the residual symptoms of MDD in addition to the increased risk of recurrence, and these can be costly and severe. Patients with residual symptoms have higher rates of myocardial infarction [49] and cerebrovascular accidents [50], and overall have a worse prognosis of their medical conditions and increased utilization of medical services [11]. 

Another common residual symptom is low interest in work [51], and patients with incomplete remission of MDD, as compared with full remitters, had higher levels of absenteeism, lower productivity, greater interpersonal difficulties, and lower job satisfaction [52]. The cost of major depression in the U.S. in 1990 was estimated to be $44 billion; missed days of work and lower productivity account for approximately $24 billion of that sum [53]. Patients with residual symptoms are more than twice as likely to require public assistance benefits compared to those with no residual symptoms [19]. The ability to participate in leisure activities and relationships are also impaired in patients with residuals symptoms of MDD, and it is not surprising that marital relationships suffer as well [54]. There is some evidence that if the initial episode of MDD depression is not severe, such functional impairments are more likely to resolve, even in cases where residual core symptoms of MDD persist for extended periods of time [17]. 

In addition to the persistent symptoms and functional impairments, there may be long term physiologic changes in patients with residual symptoms of MDD. These patients are at increased risk of developing treatment-resistant depression [55], and there is evidence that chronic or recurrent major depression is associated with morphometric brain changes, including bilateral reduction in hippocampal volume [56].

Though the underlying neurobiological dysfunctions in both MDD and in the residual symptoms of MDD remain to be fully elucidated, potential biological factors associated with residual symptoms may include genetic variability of CYP450 genes, expression of brain-derived neurotrophic factor (BDNF), or serotonin 5HT transporter density, as well as underlying disturbances in the hypothalamic-pituitary-adrenal (HPA) axis [57]. Any of these factors could cause certain patients to have an increased susceptibility to ongoing residual symptoms and recurrence of depression in a manner that is refractory to our current treatment modalities. The theoretical possibility exists for assessing patients’ underlying biologic vulnerabilities and for targeting treatments accordingly [58], but the search for such definitive biomarkers has, to date, yielded few practical results [59].

## 3. Treatment of Residual Symptoms

**Figure 3:**
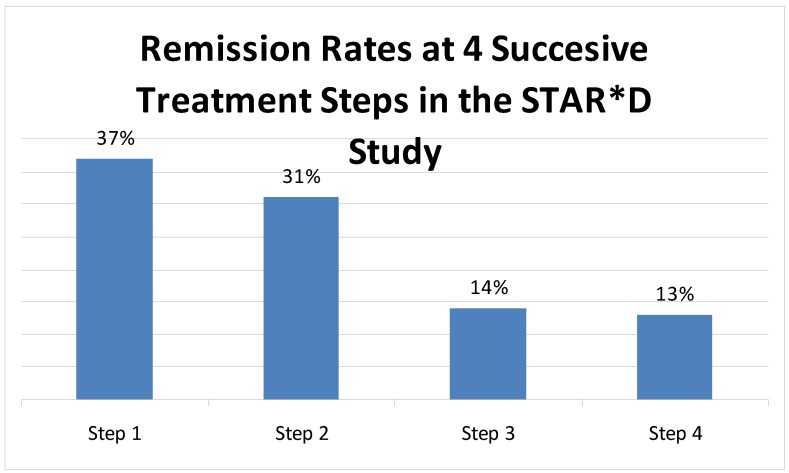
Sequential remission rates at each step in the STAR*D study.

An interesting point of view has been put forward that suggests that some patients with a treatment-refractory depression would be best managed by aiming as much as possible to reduce symptomatology, while considering their mood disorder to be a chronic condition without expectation for full recovery. This perspective emphasizes symptom management and quality of life rather than vigorous pharmacotherapy, with the potential advantages of avoiding the unnecessary costs and side effects as well as the patient demoralization that can be associated with repeated failed polypharmacy [60]. One data point supporting this view is that despite pharmacologic and psychotherapeutic advances over the last 20 years, including the introduction of many new antidepressants, recurrence rates of MDD have not improved concurrently [61,62]. However, in the STAR*D study, though the cumulative remission rate was 67%, it is notable that 13% of the patients achieved remission only at the fourth sequential step in treatment [8] (Figure 3).

This is a small but meaningful number and supports the practice of continuing successive treatments attempts in patients whose depression at first appears refractory to treatment. This is particularly important since these patients with residual symptoms are so vulnerable to recurrence of MDD and all the associated sequelae thereof [63].

In beginning treatment for residual symptoms, it can be a useful first step to “Run the Axes” – that is, to review a patient's Axis I-IV issues [64]. Axis I: is the initial diagnosis correct? Could there be occult substance abuse? Axis II: is there are a personality disorder, such as borderline personality disorder, in which a chronic feeling of emptiness has been misconstrued as MDD? Axis III: is there a complicating medical condition impeding treatment response? Axis IV: does the patient have an ongoing life stressor, such as unemployment or divorce, which can limit the benefit that can be expected from pharmacology alone [65] (Table 1 [64]).

Compliance should also be considered before modifying a treatment regimen, as up to 20% of treatment resistance can be attributed to poor compliance with recommended interventions [66]. Other steps that should be taken prior to initiating treatment for any patient with residual symptoms include proper psychoeducation and expectation setting with patients [70], and insuring adequacy of dosage [67] and appropriate duration of treatment [16]. 

**Table 1:** Running the Axes [64].

“Run the Axes:” Factors to Consider Before Modifying a Treatment Plan for Major Depressive Disorder	
Axis I:	Is the initial diagnosis correct?
Axis II:	Could there be occult substance abuse?
	Is there are a personality disorder that is either co-morbid with, or that appears similar to, a mood disorder?
Axis III:	Is there a complicating medical condition impeding treatment response?
Axis IV:	Does the patient have an ongoing life stressor that may limit the benefit that can be expected from pharmacology alone?

There are insufficient controlled studies of pharmacotherapy that are able to provide any clear treatment steps to specifically address residual symptoms of MDD. Most of what is used in clinical practice tends to be extrapolated from trials that do not address residual symptoms of MDD, but rather MDD that is treated to the generally accepted standard of remission; however, as has been established, this definition is not based on bringing patients to a completely symptom-free state and can include varying degrees of ongoing depressed mood and other impairments. There is somewhat more available guidance on the application of psychotherapy to treat residual symptoms than there is for medications, with both traditional Cognitive Behavioral Therapy (CBT) and mindfulness-based CBT, which emphasizes meditation, providing positive treatment outcomes [48,68].

The primary pharmacotherapy strategies available to treat residual symptoms are: further time on the same medication [16], switching medications [69], sequential treatments (primarily using medications followed by psychotherapy) [48] and augmentation or combination with additional medications [70]. It has even been suggested that since the rates of complete remission from monotherapy are so low, combination or augmentation treatments should be considered as a first-line pharmacotherapy strategy [70]. 

## 4. Augmentation/Combination

Lithium is the medication with the longest record of efficacy in treating partial response to monotherapy, though much of the evidence supporting its usage as an augmenting agent pertains to augmentation of tricyclic antidepressants (TCAs) and monoamine oxidase inhibitors (MAOIs) [71,72]. The benefit of lithium when combined with the selective serotonin reuptake inhibitors (SSRIs) and serotonin-norepinephrine reuptake inhibitors (SNRIs) is not as well established [70]. However, in the STAR*D study, the addition of lithium to SSRIs and SNRIs did show benefit in a small percentage of patients whose depression was otherwise refractory to treatment [73]. Despite the probable benefits, the popularity of lithium is limited by its narrow therapeutic window with potential for toxicity and the need for monitoring serum levels [70]. 

L-Triiodothyronine (T3) has also been best studied as an augmentation agent of the TCAs and MAOIs [74], with few controlled studies investigating its benefit in combination with newer antidepressants [73]. It may be that T3 augmentation is better for accelerating the response to treatment rather than enhancing likelihood of remission [73]. 

The most commonly used combination agent is bupropion [75], and though its frequency of such usage probably exceeds the available evidence, there is some support for its role as a combination agent [76]. There are several likely reasons why bupropion is such a common combination agent: it is generally a well-tolerated medication [77], it can provide some benefit in counteracting the sexual side effects of the SSRIs, and its presumed mechanism of action as an indirect norepinephrine agonist and dopamine reuptake blocker is, in theory, an attractive accompaniment to the action of SSRIs [78]. After bupropion, the next most common combination agent is mirtazapine [75]. Mirtazapine has noradrenergic activity as well as serotonergic activity mediated through a different mechanism of action than the SSRIs [70], and its adverse side effect profile of weight gain and sedation can provide relief to patients with loss of appetite and insomnia [70]. As with bupropion, there is not a great deal of confirmation from controlled studies regarding the usage of mirtazapine as a combination agent, but what data exist does provide some supporting evidence [79,80]. At present, the theoretical basis for a complementary benefit of targeting more than one neurotransmitter system, such as by adding an agent with noradrenergic or dopaminergic activity to an SSRI, remains largely conjecture. STAR*D results suggest that when switching from one antidepressant to another due to lack of remission, one antidepressant may be as effective as the next, and neither was there strong evidence regarding which augmentation agent to choose should a physician decide to augment rather than switch [81]. 

One significant treatment not included in the STAR*D algorithm was electroconvulsive therapy (ECT), so it remains unclear what the remission rates would have been for those patients whose MDD did not remit in the first four steps of treatment [82]. However, ECT is known to be effective at times in cases where pharmacotherapy has failed [83,84], and it should be considered as an important tool to help patients achieve complete remission. Other somatic neuromodulating techniques, such as repetitive transcranial magnetic stimulation (rTMS), vagus nerve stimulation (VNS) and deep brain stimulation (DBS) show varying degrees of promise in patients who do not achieve complete remission of MDD with medications or psychotherapy, but require further study and refinement before they enter general usage [85].

Other treatments with some possibility of benefit in treating residual symptoms of MDD include buspirone [69], modafinil [86], and folate [87]. Numerous studies have found some degree of benefit in treating SSRI non-responders with the atypical antipsychotics [88,89]. However, the usage of atypical antipsychotics must be considered carefully, given the clear evidence of their role in causing serious weight gain, hyperlipidemia and metabolic disturbances [90].

## 5. Conclusions

The current definition of remission from depression neither fully takes into account all aspects of patient wellness, nor the presence and risks of residual symptomatology. Treating patients to a state where they no longer meet criteria for Major Depressive Disorder must often be considered only a starting point to treatment. Though there is not clear guidance in the medical literature as to how best to treat residual symptoms, there is a convincing body of evidence indicating why clinicians should try.
